# Fluid outflow in the rat spinal cord: the role of perivascular and paravascular pathways

**DOI:** 10.1186/s12987-018-0098-1

**Published:** 2018-04-29

**Authors:** Shinuo Liu, Magdalena A. Lam, Alisha Sial, Sarah J. Hemley, Lynne E. Bilston, Marcus A. Stoodley

**Affiliations:** 1 Department of Clinical Medicine, Faculty of Medicine and Health Sciences, Macquarie University, Suite 407, Clinic Building, 2 Technology Place, Sydney, NSW 2109 Australia; 2 Neuroscience Research Australia, Prince of Wales Clinical School, University of New South Wales, Margarete Ainsworth Building, Barker Street, Randwick, Sydney, NSW 2031 Australia

**Keywords:** Cerebrospinal fluid, Outflow, Spinal cord, Syringomyelia, Perivascular, Interstitial fluid

## Abstract

**Background:**

Cerebrospinal fluid (CSF) is thought to flow into the brain via perivascular spaces around arteries, where it mixes with interstitial fluid. The precise details concerning fluid outflow remain controversial. Although fluid dynamics have been studied in the brain, little is known about spinal cord fluid inflow and outflow. Understanding the normal fluid physiology of the spinal cord may give insight into the pathogenesis of spinal cord oedema and CSF disorders such as syringomyelia. We therefore aimed to determine the fluid outflow pathways in the rat spinal cord.

**Methods:**

A fluorescent tracer, Alexa-Fluor^®^-647 Ovalbumin, was injected into the extracellular space of either the cervicothoracic lateral white matter or the grey matter in twenty-two Sprague–Dawley rats over 250 s. The rats were sacrificed at 20 or 60 min post injection. Spinal cord segments were sectioned and labelled with vascular antibodies for immunohistochemistry.

**Results:**

Fluorescent tracer was distributed over two to three spinal levels adjacent to the injection site. In grey matter injections, tracer spread radially into the white matter. In white matter injections, tracer was confined to and redistributed along the longitudinal axonal fibres. Tracer was conducted towards the pial and ependymal surfaces along vascular structures. There was accumulation of tracer around the adventitia of the intramedullary arteries, veins and capillaries, as well as the extramedullary vessels. A distinct layer of tracer was deposited in the internal basement membrane of the tunica media of arteries. In half the grey matter injections, tracer was detected in the central canal.

**Conclusions:**

These results suggest that in the spinal cord interstitial fluid movement is modulated by tissue diffusivity of grey and white matter. The central canal, and the compartments around or within blood vessels appear to be dominant pathways for fluid drainage in these experiments. There may be regional variations in fluid outflow capacity due to vascular and other anatomical differences between the grey and white matter.

## Background

Details of the circulation of cerebrospinal fluid (CSF) and interstitial fluid (ISF) of the central nervous system remain controversial [1, 2]. In recent decades, the concept of CSF circulating through the brain parenchyma, as a mechanism for metabolite transport and clearance [1–4], has gained momentum. ISF consists of water and solutes that are the by-products of cellular metabolism and synaptic transmission in the extracellular space. There may even be a component of ISF that passes across the brain capillary endothelium (although compelling in vivo evidence is contentious) [4–8]. Perivascular spaces have received renewed interest as a crucial facilitator of fluid inflow in neural tissue [9–13]. If CSF can enter brain parenchyma, there must also be efflux pathways [3]. The assumption is that ISF must be cleared, probably into the subarachnoid space [14, 15], but the precise mechanism is unclear.

The “glymphatic” theory of fluid homeostasis posits that fluid flow into and out of the parenchyma is via arterial and venular pathways respectively [10, 16, 17]. Studies to date have largely focused on the brain, with few investigations of spinal cord. Although there is some evidence of similar mechanisms governing fluid ingress [18–20], how fluid egresses from the cord is almost unknown [21, 22]. Compared to the brain the spinal cord is not only much smaller, but the arrangement of the grey and white matter is reversed. Furthermore, spinal cord axonal tracts are oriented parallel to its long axis. These fundamental anatomical differences mean diffusion and transport of fluid in grey and white matter are likely to be different [3, 14, 23].

Syringomyelia is a puzzling condition where fluid filled cysts develop in the spinal cord, usually secondary to another pathology, such as trauma, that results in CSF obstruction in the subarachnoid space. There is emerging evidence that its pathogenesis is a dynamic process involving imbalances in fluid inflow and outflow. The important contribution of perivascular spaces to mechanisms of fluid entry into syrinxes has been characterised in previous animal experiments [19, 24, 25]. Recent work [26] on fluid outflow pathways in an ovine model of post-traumatic syringomyelia indicated diffuse fluid movement away from the syrinx cavity and towards the central canal and perivascular spaces. However, the precise pathways of fluid drainage in the spinal cord under normal physiological conditions, and whether perivascular spaces play a crucial role in this context are unknown. A more complete understanding of the mechanisms governing spinal cord fluid homeostasis may lead to new insights into the pathogenesis of syringomyelia.

In this study, we aimed to determine the fluid outflow pathways in the rat spinal cord. We injected a fluorescent tracer of the extracellular space, ovalbumin conjugated to the fluorophore Alexa-Fluor^®^-647 (AFO-647), into the spinal grey and white matter of Sprague–Dawley rats. Our hypotheses were: (1) fluid outflow from the spinal cord is via the perivenular spaces; and (2) the pattern of fluid flow in the white matter is different from that of grey matter.

## Methods

Ethics approval was obtained from Macquarie University Animal Ethics Committee (ARA 2016/032–5). Outflow from the grey and white matter was separately investigated at two time points in 22 male Sprague–Dawley rats, weighing from 155–345 g. Ten animals were used in the white matter injection studies, while 12 were used in the grey matter injection studies.

### Surgical procedure

After induction of general anaesthesia with 4% isoflurane in oxygen, the animal was positioned prone in a stereotactic frame, and maintained under anaesthesia with 2.5% isoflurane (adjusted as necessary) in 0.2 L/min of oxygen. Heart rate, oxygen saturation, respiratory rate and rectal temperature were continuously recorded.

Under an operating microscope, a dorsal midline occipitocervical incision was made followed by subperiosteal muscle dissection. Segmental laminectomies at C7/T1 or T1/T2 were performed with fine rongeurs. A window of thecal sac, eccentric to the right, was exposed. A 34G Nanofil needle, loaded onto a glass syringe (World Precision Instruments, Florida, USA), punctured the dura in a single pass. For grey matter studies, the entry point of the needle was 0.5 mm right of the midline at the C7/T1 interspace. For white matter studies the entry was at least 1 mm right of the midline at the T1/T2 interspace, where the grey matter is less prominent. The needle passed into the parenchyma to a depth of 1 mm targeting either the junction of the ventral and dorsal horns (for grey matter injections), or the lateral white matter funiculus. An Ultramicro pump (World Precision Instruments, Florida, USA) was used to deliver 500 nL of Ovalbumin Alexa-Fluor^®^-647 conjugate (Life Technologies, Victoria, Australia) with 10% fluorescent microspheres (v/v %) (Thermo Fisher Scientific, Massachusetts) at a rate of 2 nL/s. The needle was left in situ for either 20 or 60 min from the start of the injection. By either the 20 or 60 min time point, the animal underwent transcardiac perfusion with heparinised 0.1 M phosphate buffered saline (PBS) followed by 4% paraformaldehyde (PFA) (Lancaster Synthesis, Pelham, New Hampshire).

### Tissue processing

The spinal cord and brain were harvested en bloc for macroscopic fluorescent imaging. After post fixation in 4% PFA overnight, the specimen was stored in 30% sucrose for cryoprotection. The spinal cord was segmented from C2 to T4. Each segment was snap frozen, and 40 µm axial sections were taken on a cryostat and mounted onto glass slides.

### Immunohistochemistry

The glass slides were washed twice for 10 min in tris–phosphate buffered saline, and then in 50% ethanol for cellular permeabilization. After application of 15% normal donkey serum (NDS) blocking solution, the slides were incubated overnight with 1:100 Rat Endothelial Cell Antibody (RECA-1, Abcam, Cambridge, United Kingdom) in 4% NDS. The secondary antibody, 1:400 anti-mouse IgG Alexa-Fluor^®^-488 (Molecular Probes, Life Technologies, New York, USA) was then applied. This was followed by anti-actin α-smooth muscle antibody at 1:400 dilution (SMA-Cy3, Sigma-Aldrich, St. Louis, Montana). Primary and secondary controls were established to exclude autofluorescence. The slides were cover-slipped with fluorescent mounting medium (DAKO, NSW, Australia).

### Image acquisition

After post fixation, macroscopic white-light and single channel fluorescent images were captured with the in-vivo MS FX PRO (Bruker, Billerica, MA). The fluorescence camera was set at excitation and emission wavelengths of 630 and 700 nm respectively, with an exposure time of 4 s.

Spinal cord axial sections from C2 to T4 were imaged with a Zeiss Axio Imager Z1 fluorescence microscope (Carl Zeiss Microimaging GmbH, Germany) for qualitative and quantitative analysis. The fluorescent microspheres, which have a diameter of 1 μm, were used to verify the location of the injection site as their size prevents significant displacement. SMA- and RECA-1-positive vessels were identified as arterioles. SMA-negative, RECA-1-positive vessels were designated venules or capillaries. Those with largest diameter ≥ 6.5 µm were considered venules, and those < 6.5 µm capillaries. Further delineation of vascular and anatomical structures was undertaken with confocal microscopy (LSM 880, Carl Zeiss Microimaging GmbH, Germany).

### Image analysis

**Fig. 1:**
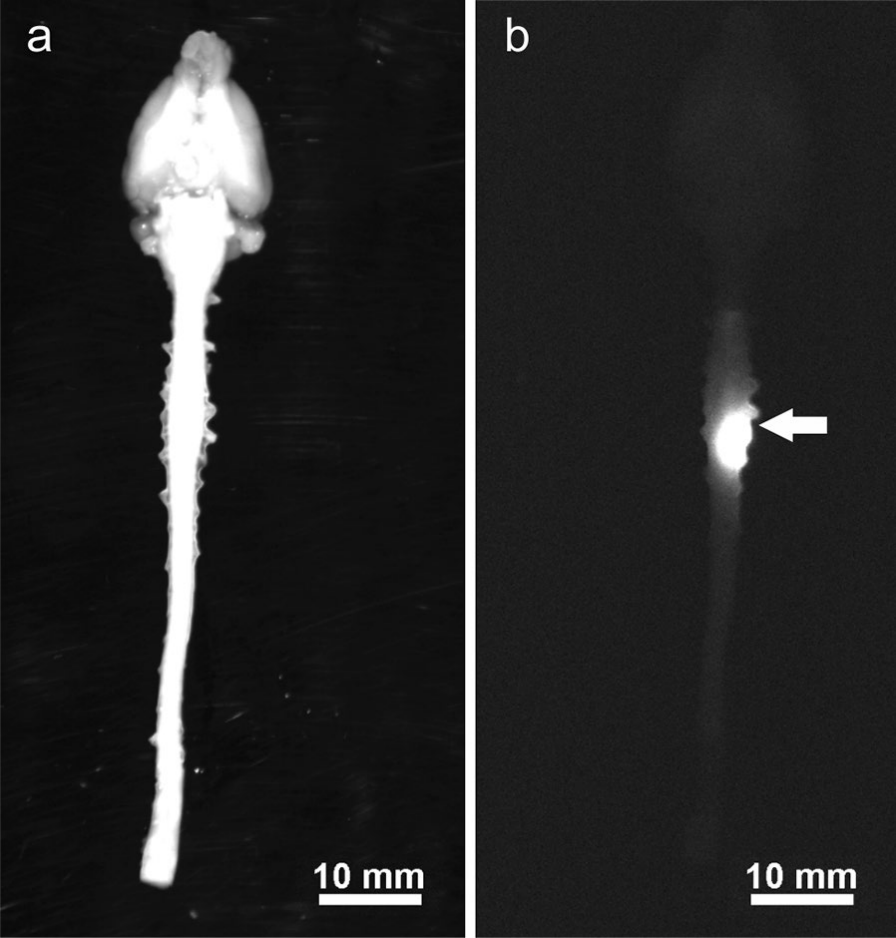
White light and single fluorescence channel acquisition of harvested brain and spinal cord with the in-vivo MS FX PRO Multispectral Imager System. Brightness and contrast have been uniformly adjusted for optimal visualisation. **a** White light enabled spinal level localisation. **b** Macroscopic appearance of tracer distribution. There is a sharp drop off in fluorescence intensity within 1–2 spinal levels rostral and caudal to injection site at C7/8 (arrow)

Quantitative analysis of fluorescent signal intensity was performed using Image J, version 1.46r [27]. Subtraction of background fluorescence was performed in all measurements. In macroscopic fluorescent acquisitions, the spinal segment levels were identified by counting nerve roots on the white light images (Fig. 1a). These were then overlaid onto the fluorescence images. Mean pixel densities were measured in each spinal segment from C2 to T4 to yield fluorescence intensities.

In fluorescent photomicrographs of axial sections, the integrated density of the tracer (mean pixel density multiplied by area) was calculated. The mean pixel densities of the white and grey matter were measured separately. At least three sections were analysed per spinal level from C2 to T4, and then averaged to give a mean integrated density.

### Statistical analysis

Grey matter and white matter integrated densities were compared using two-way analysis of variance (ANOVA) and adjusted for multiple comparison using Bonferroni’s post hoc tests (GraphPad Prism v7.02, GraphPad Software Inc, California). A *p* value < 0.05 was considered statistically significant. All values were expressed as mean ± standard error of the mean (SEM).

## Results

### Rostral–caudal tracer distribution

**Fig. 2:**
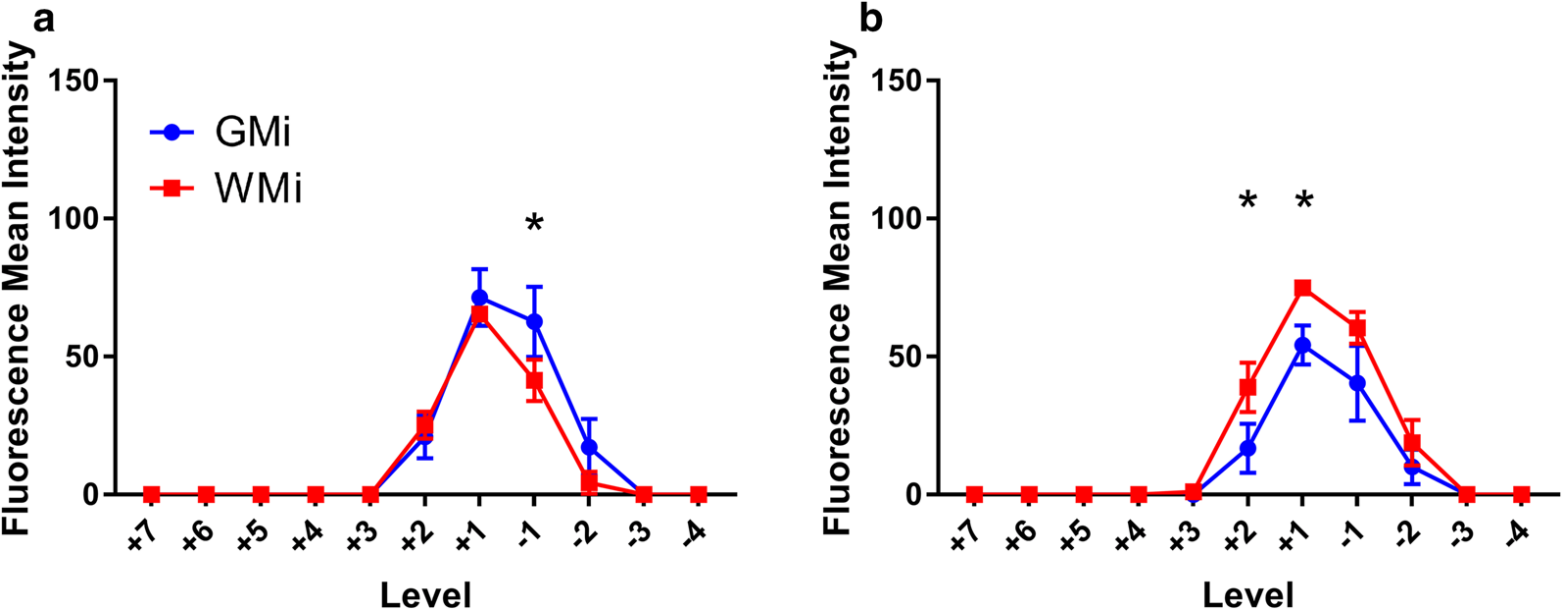
Quantification of rostral–caudal tracer fluorescence (mean fluorescence intensity) per spinal level after grey (n = 10) and white (n = 10) matter injections at 20 min (**a**, left panel) and at 60 min (**b**, right panel). Each spinal cord level (“Level”) is expressed as the number of levels rostral (positive integers) or caudal (negative integers) to injection site. All error bars are expressed as ± SEM. In both white and grey matter injections at both time points, there was a sharp drop off of tracer fluorescence within 2 levels rostral and caudad to the injection. At the 20 min time point **a**, there was no difference in fluorescence intensity between white and grey matter injections, but on post hoc analysis a significant difference was reached at − 1 level caudal to the injection site (*p = 0.0341). At the 60 min time point **b**, fluorescence intensity was significantly higher in the white matter injections compared to the grey matter injections (p = 0.0026). On post hoc analysis significant differences were observed at + 1 and + 2 levels rostral to the injection point (*p = 0.0448 and 0.0259 respectively)

In macroscopic fluorescent imaging, tracer was observed to be localised to the injection site in all experiments (Fig. 1b). The macroscopic mean fluorescence intensity was determined for each spinal cord level. A sharp drop-off in intensity within two levels rostral and caudal to the injection site was observed (Fig. 2). At the 60 min time point, but not at the 20 min time point, the cord had significantly higher mean fluorescence intensities after white matter injections (WMi) compared to grey matter injections (GMi) (two way ANOVA, p = 0.0026). On post hoc analysis, significance was reached one and two levels rostral to the injection point (p = 0.045 and 0.026 respectively) (Fig. 2b). Post hoc analysis also demonstrated a significant difference between white and grey matter injections at the 20 min time point one level caudal to the injection site (p = 0.034) (Fig. 2a).

### Axial tracer distribution

**Fig. 3:**
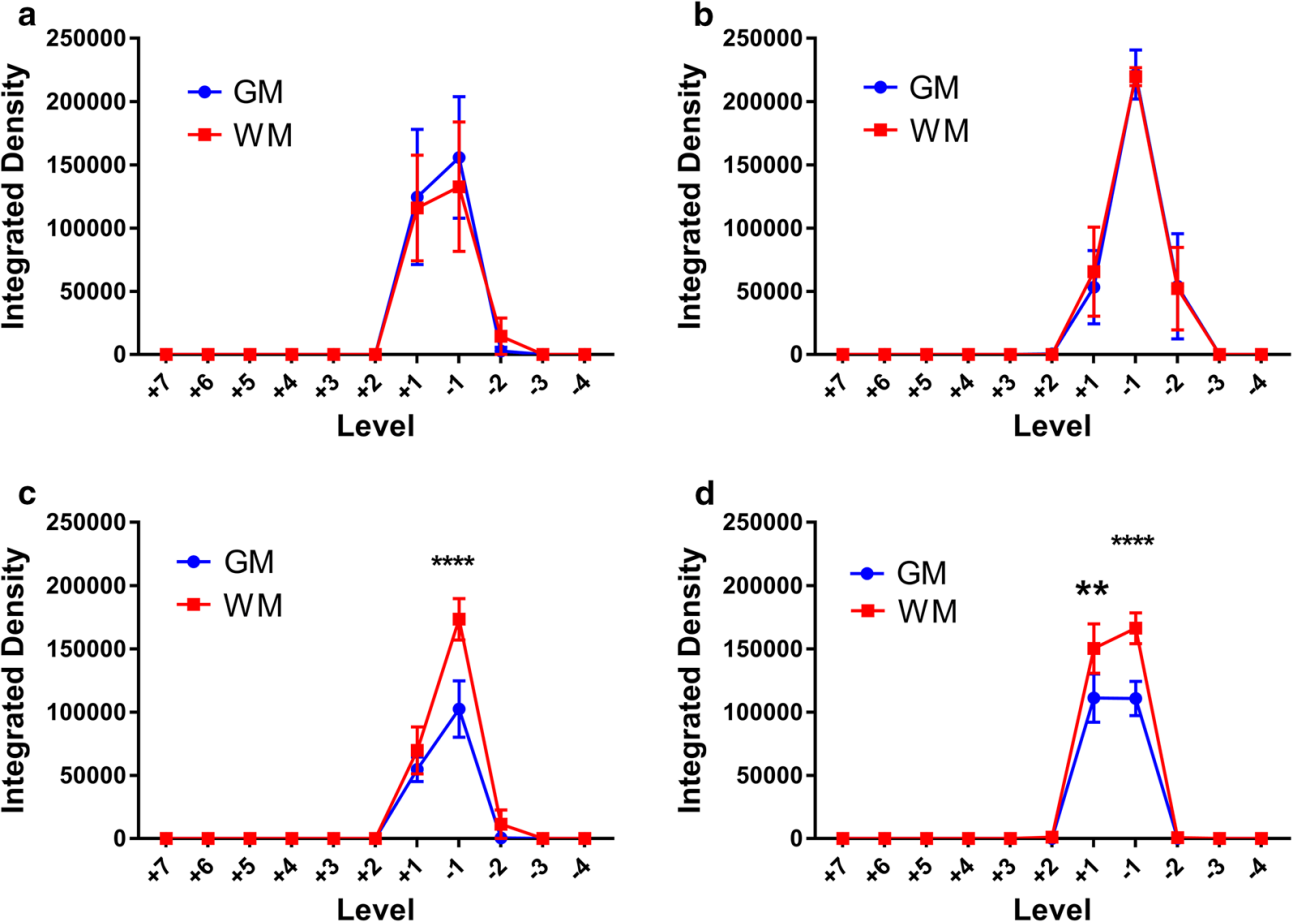
Quantification of microscopic axial section tracer fluorescence (integrated density) per spinal level after grey and white matter injections. Each spinal cord level (“Level”) is expressed as the number of levels rostral (positive integers) or caudal (negative integers) to injection site. All error bars are expressed as ± SEM. **a** After grey matter injections at 20 min (n = 5) there was no statistical difference between grey and white matter fluorescence. **b** This was also observed in grey matter injections after 60 min (n = 5). However, after white matter injections at **c** 20 min (n = 5) and at **d** 60 min (n = 5), there was significantly greater tracer fluorescence in the white matter compared to the grey matter (p = 0.0094 and 0.0041 for 20 and 60 min respectively). On post hoc analysis, a statistically significant difference was observed at one level caudal to the injection site (***p < 0.0001) at 20 min (**c**), and one level rostral and caudal at 60 min (**d**) (**p = 0.0017, ****p < 0.0001)

Quantification of AFO-647 intensity from microscopic analysis of axial sections is summarised in Fig. 3a–d. The fluorescence intensity within the grey and white matter, expressed as integrated density, is represented in relation to spinal level at the 20 and 60 min time points separately. After white matter injections, at both 20 and 60 min, fluorescence was significantly greater in the white matter compared to grey matter (p = 0.0094 and 0.0041 for 20 and 60 min respectively) (Fig. 3c, d). On post hoc analysis, at the 20 min time point, a significant difference was observed at one level caudal to the injection site (p < 0.0001). At 60 min, white matter fluorescence was found to be significantly greater one level rostrally (p = 0.0017) and caudally (p < 0.0001). Following grey matter injections, however, tracer fluorescence was not significantly different between grey and white matter, at either time point (Fig. 3a, b).

### Pattern of tracer distribution: grey matter injections

**Fig. 4:**
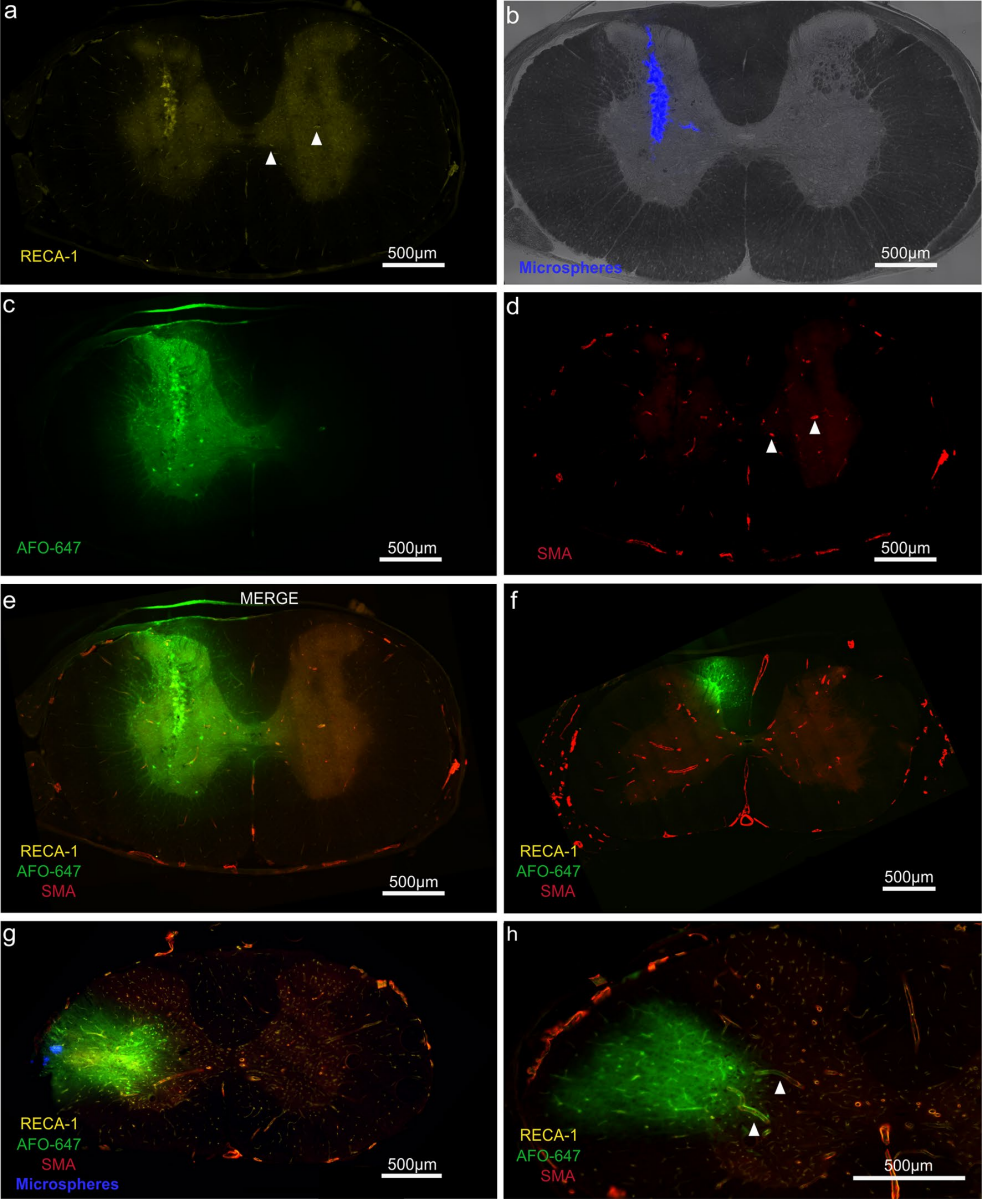
Typical axial sections at the cervicothoracic junction after injection of fluorescent tracer into the spinal grey and white matter. **a**–**e** Grey matter injection. **a** RECA-1 and **d** SMA immunofluorescent staining of arterioles. Examples of grey matter arterioles are marked by arrow heads in **a**, **d**. Arterioles were present in greater numbers in the grey matter compared to white matter. **b** Fluorescent microspheres confirmed the Nanofil needle had traversed the grey matter. **c**, **e** Radial redistribution of tracer from the middle of the grey matter in all directions. **f** Axial section rostral to a grey matter injection site where a significant amount of tracer had spread into dorsal column. Note tracer fluorescence was mainly confined to the dorsal white matter column at this level. **g** After delivery into the white matter, AFO-647 tracer conformed to the shape of the lateral funiculus with limited spread into the grey matter. **h** In rostral sections in the same animal, tracer was confined to the white matter. Arrow heads demonstrating selective tracer deposition around arterioles. All fluorescent photomicrographs were taken at ×20 magnification

In six of 12 animals, in which grey matter injections were performed, tracer was delivered to the junction of the ventral and dorsal horns. There was a continuous radial decrease in fluorescence intensity in all directions away from the injection site. Tracer signal was detected in the white matter surrounding the grey matter at the injection level (Fig. 4e). In rostral and caudal axial sections, tracer was detected predominantly in the grey matter.

In the other six animals, in which grey matter injections were performed, tracer was delivered into either the middle of the ventral or dorsal horn. Although the highest fluorescence intensity was found within the grey matter, there was substantial tracer signal in the adjacent white matter. In rostral and caudal sections there was prominent tracer signal in the white matter (Fig. 4f). In all but one animal, tracer was detected in the contralateral grey matter.

### Pattern of tracer distribution: white matter injections

In eight of 10 animals the distribution of AFO-647 conformed to the shape of the lateral funiculus, staying primarily in the white matter (Fig. 4g). A radial reduction in tracer fluorescence was also observed. A small amount of tracer entered the lateral horn of the grey matter. Rostrocaudally, ovalbumin was almost exclusively found in the white matter (Fig. 4h). Within this subgroup of animals, no tracer was detected in the contralateral grey matter except in one animal.

In two of 10 animals, there was a similar pattern of tracer spread in the white matter but considerable ovalbumin also redistributed into the grey matter. Rostrocaudally, however, tracer was confined to the white matter.

### Tracer in relation to vascular structures

**Fig. 5:**
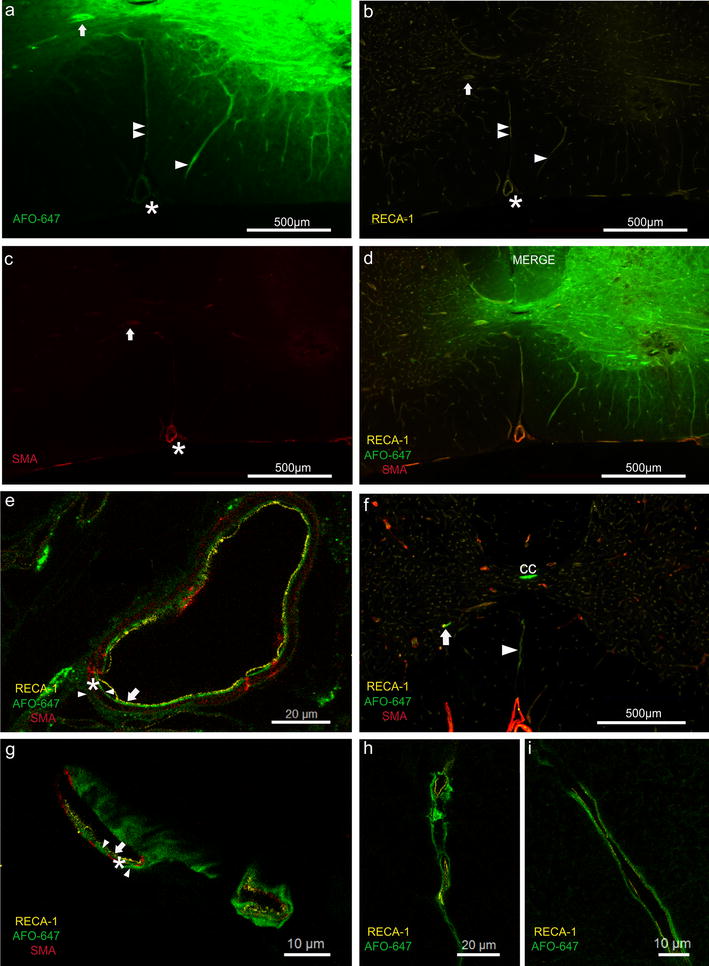
Relationship of injected tracer to vascular structures. **a**–**d** Fluorescent microscopy of grey matter injection. Tracer co-localised with the wall of the anterior spinal artery (asterisk). A radially directed venule (single arrow head) and veins (note RECA-1 positive and SMA negative) in the ventral median sulcus (double arrow heads) appeared to conduct ovalbumin away from the injection site towards the pial surface. Prominent accumulation of tracer around an arteriole (marked by arrow) against a relatively low background fluorescence suggests it is a pathway for fluid outflow. **e** Confocal photomicrograph of the anterior spinal artery found in **d**. A layer of AFO-647 tracer (indicated by right pointing arrow head) was detected external to the tunica media (SMA positive, indicated by asterisk). Another distinct layer of fluorescent tracer was also found internal to the tunica media layer (left pointing arrow head), separate from the endothelial layer (RECA-1, marked by arrow). **f** Pronounced tracer deposition around a “remote” arteriole (arrow) and vein in the ventral median sulcus (arrow head). These vessels were one level rostral to the grey matter injection site, and therefore tracer accumulation around these structures could not be explained by contiguous tracer spread. It is likely ovalbumin was transported over a distance in the spaces around these vessels. Note tracer labelling of the central canal (indicated by “cc”). **g** “Peri- and para-arterial” pattern of tracer deposition in specific compartments external and internal to the tunica media of parenchymal arterioles (arrow heads, arrow and asterisk denote the same anatomical layers as in **e**). **h** Tracer accumulation between the adventitia and the glia limitans of veins in the ventral median sulcus (found in **f**). **i** The same “para-venular” pattern demonstrated in a radially directed parenchymal venule, found in **d**. All fluorescent and confocal photomicrographs were taken at ×20 and ×63 magnification respectively

**Fig. 6:**
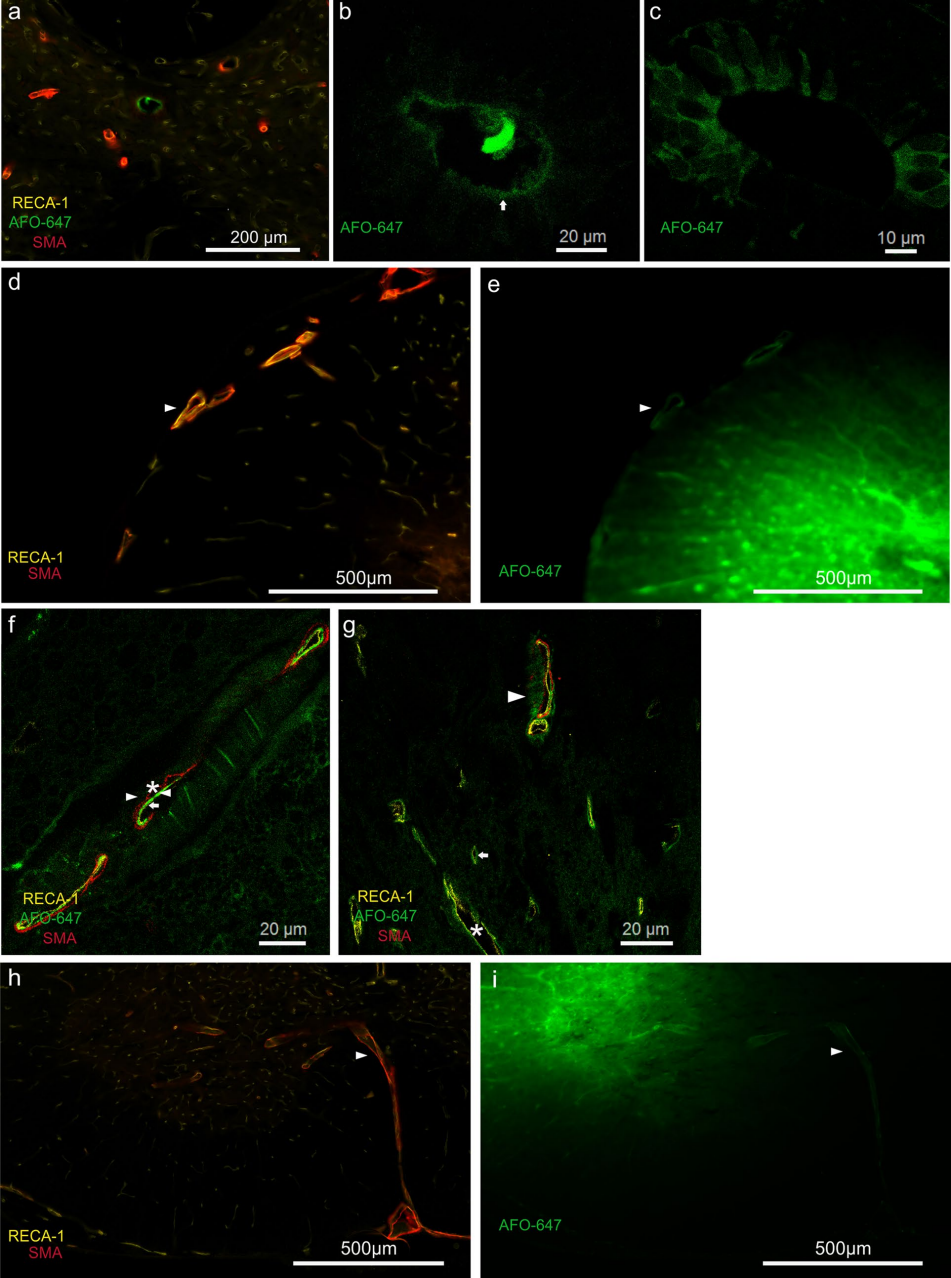
Tracer delivered into the spinal cord parenchyma accumulated around ependymal and extramedullary structures. Fluorescent (**a**) and confocal (**b**) micrographs demonstrating tracer accumulation in the central canal. Note the presence of tracer within the lumen in **b** (12 o’clock position). **c** Confocal microscopy of central canal in another experiment. The ependymal cells were heterogeneously delineated by fluorescence, with the noted absence of nuclear tracer signal. In both **b** and **c**, the apical ends displayed greater tracer intensity compared to the basal surface. **d**, **e** Tracer deposition around the arterial vasocorona (arrow heads, note RECA-1 and SMA positivity) of the dorsal spinal cord surface. **f** Confocal microscopy view of the same arterial vasocorona demonstrating the characteristic “peri-arterial” and “para-arterial” distribution of the tracer (arrow heads) with respect to the tunica media (asterisk) and endothelium (arrow). The absence of subpial tracer signal excludes the possibility of contiguous tracer spread from injection site to artery. The arterial vasocorona could be the dominant pathway for fluid outflow from the white matter. **g** Fluid outflow appeared to involve all vascular structures. Confocal microscopy of grey matter showing arteriolar (arrow head), venular (asterisk) and capillary (arrow) labelling by tracer. Note the “paravascular” location of tracer in venules and capillaries. **h**, **i** Fluorescent microscopy of grey matter injection demonstrating conduction of tracer along the central branch of the anterior spinal artery towards the ventral median fissure. This suggests drainage of interstitial fluid towards the pial surface via vascular structures. All fluorescent and confocal photomicrographs were taken at ×20 and ×63 magnification respectively

In all animals fluorescent tracer accumulated around or within the walls of arterioles, venules and capillaries in both the grey and white matter (Figs. 5, 6g). Arterioles were more numerous in the grey matter than the white matter (Fig. 4d). Selective tracer labelling of vascular structures was particularly evident in areas of low background tracer concentration (Fig. 5a, d). In the white matter, tracer concentrated along arterioles and venules that extended from the grey matter to the pia (Fig. 5d).

Tracer co-localised with arterioles and venules of the ventral median fissure in all but one animal (a white matter injection, sacrificed at 20 min) (Fig. 5d, f, h). Tracer was present in the wall of the anterior spinal artery (ASA) and its central branch in 10 animals (Fig. 5d), of which nine were grey matter injections. Fluorescence was further present in the walls of the arterial vasocorona in 13 animals (Fig. 6d–f), of which 10 were white matter injections. Confocal microscopy demonstrated tracer deposition external to the smooth muscle layer of the ASA. Additionally, there was a distinct layer of tracer between the endothelial and smooth muscle layers (Fig. 5e). This pattern of tracer distribution was also observed in parenchymal arterioles and other extramedullary arteries, such as the central branch of the ASA and the arterial vasocorona. AFO-647 was discretely deposited external to the endothelial layer of capillaries and venules of the cord parenchyma (Figs. 5h, i, 6g).

In at least six animals (two from white matter injections), tracer deposited prominently around “remote” arterioles (Fig. 5f, g). These labelled vessels were far removed from the bulk of the contiguous tracer at the injection site. Tracer labelling of the pia and subpial space was generally limited or absent as fluorescence intensity decreased from the site of injection towards the cord surface. Instead, ovalbumin concentrated around vessels that traversed the cord parenchyma towards the pial surface. Tracer appeared to be transported from the injection site to the extramedullary vasculature (Fig. 6i), along these conduit-like arterioles and venules.

### Central canal

Fluorescent tracer was detected in the central canal ependymal cell layer in 6 of 12 grey matter injections. In three animals, central canal tracer fluorescence was present in at least eight contiguous spinal levels, rostral to the injection site. Furthermore, tracer was observed within the lumen of the canal, confirmed by confocal microscopy. The bordering layer of ependymal cells was heterogeneously delineated by fluorescence. Nuclear labelling by tracer was absent. The apical ends displayed greater tracer intensity compared to the basal surface (Fig. 6a–c). In two animals, central canal ependymal tracer was detected rostrally over only 2 spinal levels. In one animal, tracer extended caudally only from T1 to T4. No tracer was found in the central canal in any of the white matter injection animals.

### Effect of time

**Fig. 7:**
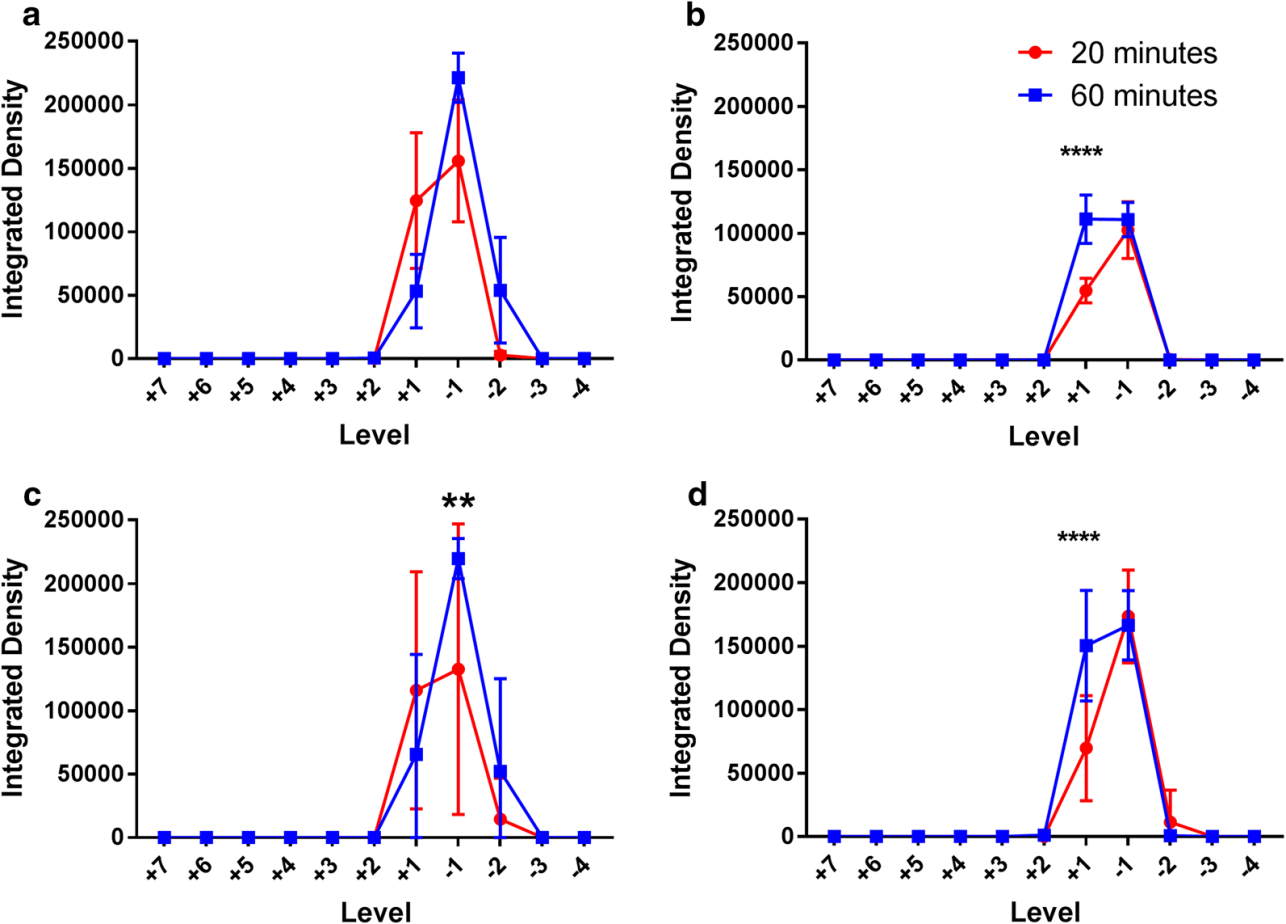
Comparison of tracer fluorescence (integrated density) in axial sections at the 20 and 60 min time points per spinal level to assess the effect of time on tracer distribution. Each spinal cord level (“Level”) is expressed as the number of levels rostral (positive integers) or caudal (negative integers) to injection site. All error bars are expressed as SEM. **a** After grey matter injections, no statistical significant difference between the time points was observed in the fluorescence intensity in the grey matter. **b** Following injection of tracer in the white matter, no statistically significant difference was observed between the 20 and 60 min groups in the grey matter. However, on post hoc analysis there was significantly greater fluorescence at + 1 level rostral to the injection site after 60 min (****p < 0.0001). Similarly, after both **c** grey matter injections and **d** white matter injections, there was no overall statistical significant difference between the 20 and 60 min groups in the white matter. However, post hoc analysis demonstrated greater integrated densities at 60 min (compared to 20 min) − 1 level caudal (**p = 0.009) and + 1 level rostral (****p < 0.0001) to the injection site in **c** grey matter and **d** white matter injections respectively

Figure 7a–d compares tracer fluorescence intensity in both the white and grey matter at the 20 min time point to that of the 60 min group. The same data derived from the quantification of axial tracer fluorescence was used to assess the effect of time on tracer distribution. There was no statistically significant difference in the grey matter fluorescence intensities between the two time points after either grey or white matter injection (Fig. 7a, b). However, on post hoc analysis significantly greater grey matter fluorescence was observed at one spinal level rostral to the white matter injection site after 60 min compared to 20 min (p < 0.0001). There was no overall significant difference in the white matter fluorescence intensities between the two time points after either grey or white matter injections. Post hoc analyses demonstrated significantly higher white matter fluorescence at 60 min compared to 20 min at one level caudal (p = 0.009) and one level rostral (p < 0.0001) to the injection site following grey matter and white matter injections respectively (Fig. 7c, d). At the longer time point, it appeared that after white matter injections there was greater redistribution of tracer from the white matter into the grey matter, and also along white matter tracts rostrally. After grey matter injections, there also appeared to be greater tracer spread into the lateral white matter caudally with time.

## Discussion

In this study, the distribution of fluorescent tracer up to 60 min after injection into the spinal cord interstitium was limited to the adjacent two to three spinal cord levels. Tracer was distributed in a radial pattern after delivery into the grey matter, with dissemination into white matter. The absence of statistically significant differences between tracer fluorescence intensities of the grey and white matter after grey matter injections (Fig. 3a, b) is consistent with this observation. However, there was limited redistribution of tracer from white into grey matter after white matter injections. The statistically significant differences between grey and white matter tracer fluorescence intensities after white matter injections support this observation. There was some evidence these patterns were amplified over time. Greater spread of tracer along white matter tracts longitudinally was also observed. There was prominent labelling of all vascular structures by AFO-647. Tracer appeared to be conducted away from the injection site towards the pial surface by depositing around radially projecting arterioles and venules. Support for this inference was provided by the detection of tracer fluorescence around extramedullary vessels. This finding was unlikely to have been secondary to diffusion (or other means of contiguous solute transport such as bulk flow) of tracer because of the general absence of subpial fluorescence (Fig. 6e, i), particularly after grey matter injections. Further microscopic analysis revealed accumulation of ovalbumin both in the perivascular and paravascular spaces of arterial vessels, which will be discussed below.

Although it was not possible in this study to conclude whether diffusion or bulk flow governed interstitial tracer movement, our findings are in concordance with theoretical and animal models of spinal ISF movement from other groups. Confinement of tracer to white matter tracts is characteristic of anisotropic diffusion, well described in the literature on CNS diffusion tensor imaging [23], and has been confirmed in the developing rat spinal cord in ex vivo experiments [28, 29]. Here, fluid diffuses along, and is constrained by, myelinated white matter fibres that run parallel to its long axis. The unmyelinated grey matter, however, is the site of penetrating arteries and its extracellular space (ECS) is rich with somas and neurites that have no preferential orientation. Diffusion here is isotropic which may explain why in our experiments tracer in the grey matter was able to redistribute in all directions. Convection enhanced delivery (CED) studies in animal spinal cord have yielded similar findings of anisotropic movement of ligands through the white matter tracts [30, 31]. Endo et al. [21], employing Evan’s blue tracer, observed comparable results to ours but described almost no tracer penetration into grey matter after white matter injections. Moreover, Evan’s blue tracer was redistributed further rostrocaudally from the injection site in the white matter compared to the grey matter. These differences could be secondary to the larger delivered volume of tracer (2 µL) in their experiments, and the smaller molecular size of Evan’s blue compared to ovalbumin. Like other CED models and earlier ex vivo work on spinal cord ISF movement [14, 28, 30–34], in Endo’s study a large durotomy was performed, resulting in substantial CSF leak and altered hydraulic integrity of the subarachnoid space and perivascular spaces, which may in turn alter fluid inflow dynamics. Computational simulation of the rat spinal cord by Sarntinoranont et al. [35] yielded a lower hydraulic conductivity in the grey matter and thus increased tissue resistance. They showed that diffusion of macromolecules *through* ECS is limited by tortuosity (higher in grey matter) and efficacy of diffusion diminishes as the square of distance. Diffusion *along* ECS is unaffected by these factors [1, 36]. This would account for the greater penetration of tracer from grey to white matter (compared to white to grey matter) in this study, and the higher fluorescence signal rostral to the injection site observed at 60 min in white matter injections (Fig. 2b). It may also explain the apparent increase in white matter fluorescence after white matter injections at 60 min compared to 20 min, and the absence of this in the grey matter after delivery of tracer into the grey matter (Fig. 7a, d).

### Perivascular clearance

Various authors have used the terms “Virchow-Robin space”, “perivascular space” and “paravascular space” interchangeably, but also at times to refer to discrete anatomical compartments. A comprehensive review of the ultrastructure of the “perivascular” space is beyond the scope of this article, but readers are referred to excellent treatises by Bakker et al. [37] and others [3, 38–40]. For our purposes, we distinguish the “peri-arterial space”, which consists of multiple compartments within the pial sheath that accompanies the arteriole/artery as it enters the CNS parenchyma, from the “para-arterial space”. The latter is the space formed by the glia limitans and the pial sheath of the penetrating artery. The “para-venular space” is formed by the venular adventitia and the glia limitans. Collectively the “para-arterial” and para-venular” spaces form the paravascular compartment. Henceforth, “perivascular space” loosely refers to all the compartments between vessel and glia limitans. These descriptions have been derived from brain studies [2, 3, 37, 38]. Ultrastructural studies of the rat spinal perivascular space suggest similar anatomy [41].

There is compelling evidence from our study supporting the importance of the vascular basement membrane in fluid outflow in the spinal cord. Controversy still surrounds the relationship of CSF, ISF and perivascular flow in the brain. There are two prominent contemporary theories of brain perivascular flow—the “glymphatic” system [10, 42] and a vascular basement membrane model [11]. The former was borne out of experiments on transgenic mice where intraventricular, intracerebral, and intracisternal injections of CSF tracers established CSF inflow into brain via a “para-arterial” route, bulk interstitial flow, and “para-venous” outflow. Other groups later raised concerns regarding the methodology and interpretation of observations [2–4, 8, 43–46]. The Carare–Weller group has long promulgated that fluid influx is via the para-arterial space and clearance of solutes and ISF occurs via the “peri-arterial” vascular basement membrane found within the tunica media. Their model has been backed by experiments from their own laboratory [11, 38, 47, 48] and from other groups employing intravital multiphoton microscopy [49]. They also assert that at the level of the capillaries, there is adjacent bidirectional flow of fluid, with inflow occurring adjacent to the glia limitans, and outflow of ISF occurring next to the endothelium. Notably, there is no venular involvement in fluid transport. The major points of dissent are: (1) the types of vascular structure(s) that are involved in ISF and solute egress; and (2) the precise relationship of the outflowing fluid to the various compartments surrounding these vessels. In this study, confocal microscopy demonstrated the presence of tracer not only in the “para-arterial” and “para-venular” spaces, but also in the “peri-arterial” compartment. It appeared that arterioles, venules and even capillaries are implicated in fluid outflow, and hence elements of both dominant theories (that are based on brain studies) have relevance in the spinal cord. Moreover, the vascular basement membrane (as proposed by Carare–Weller) has been shown here to play an important role in solute clearance in the spinal cord, which in turn suggests ISF outflow occurs both within and outside the wall of the arteriole. While injection pump pressure could confound our interpretation of interstitial perivascular tracer deposition, it is unlikely to explain tracer accumulation around only some extramedullary vessels and “remote” arterioles that are far removed from the bulk of the tracer. Moreover, our infusion rate of 0.12 µL/min is lower than that employed by other groups [3, 50], and thus is unlikely to alter the physiological drainage pathways. Uniform perivascular distribution of tracer around the spinal pial surface, which has been previously observed following cisterna magna injections [20, 41], was not detected in our experiments. Therefore, accidental delivery into, or recirculation of tracer from the cord back into the subarachnoid space are highly improbable.

Our findings raise the possibility of a model of spinal perivascular fluid dynamics characterised by rapid bidirectional movement. Some authors have suggested that there is little or no directed net fluid displacement in the perivascular space, a concept that aligns with earlier experimental data [2, 51]. A recent mathematical modelling study proposed that although there might be fast water movement to-and-fro in the perivascular space, solute transfer is facilitated by advection or dispersion [43]. Dispersion is in turn driven by arterial pulsations, which authors of disparate theories can all agree underlie the mechanism of perivascular flow [11, 16, 18, 52]. Additionally, solutes may freely communicate between the “para-arterial space” and the “peri-arterial” space through porous barriers that have been confirmed in ultrastructural studies of the spinal cord [2, 39, 41]. If this “convection” [3] theory of bidirectional fluid displacement also applies to the “para-venous” space, then tracer molecules injected into the cord parenchyma would disperse along both arteriolar and venular pathways by way of the capillaries [11]. Initially, there is “peri-arterial” drainage of solutes via the vascular basement membrane, but tracer is then able to infiltrate the “para-arterial” space. As pulsations are much stronger in arteries, tracer is propelled further along arterioles (towards the extramedullary arteries) compared to venules. This is reflected in the preponderance of tracer around extramedullary arteries and “remote” arterioles. However, we would also expect greater “para-venular” tracer deposition at 60 min compared to 20 min. This was, however, not observed, challenging this conjecture on perivascular fluid outflow. Future studies would mandate longer time points to investigate para-venular tracer distribution.

### Central canal

Central canal labelling by tracer was detected in 50% of grey matter injection experiments, with a predilection for rostral migration. This corresponds to the earlier observation by Milhorat [22] of cephalad flow within the central canal, which gave rise to the theory that it acts as a “sink” for excess solutes and fluid from the cord interstitium. Previous work by our group and others has indicated that the central canal is a route of clearance in normal and injured rat and ovine cords [18–20, 24–26, 53, 54]. Bedussi et al. have suggested that in the brain, ISF drains preferentially towards the ependymal surface. However, this was only true in close proximity to the ependymal lining and the effect decreased away from the ventricles [15]. In the spinal cord the distance between the ependymal and pial surfaces is much smaller, so the relevance of this hypothesis is unclear. A more likely explanation for the disparate central canal labelling between grey and white matter injected animals is that diffusivity differences (which in turn are dependent on factors such as tortuosity and distance) at the grey/white matter junction result in the central canal playing a critical role in fluid outflow from the grey matter. It was not possible to clarify, based on confocal micrographs, whether tracer migration into the central canal was transcellular, paracellular or both. Further ultrastructural studies may address this.

### Clinical relevance

Findings from these experiments may contribute to a clearer understanding of various spinal cord pathologies. Although outflow pathways have been shown here to involve all vascular structures, there may be regional variations. In the spinal grey matter, there is prominent drainage of solutes and ISF via the numerous ramifying arteries from the ventral median sulcus, as well as into the central canal. In the white matter, outflow efficiency may differ as there are fewer arterioles, and greater reliance on the smaller arterial vasocorona. Extrapolating further, extra-canalicular syringomyelia (a consequence of spinal cord injuries) may be partly precipitated by pathological processes disproportionately compromising ISF drainage via white matter perivascular spaces. Outflow is unable to keep up with fluid influx, ultimately leading to fluid accumulation. Similarly, this may partially explain why spinal cord oedema preferentially follows white matter tracts, as in the grey matter there may be more robust drainage pathways.

### Limitations

As some authors have emphasized [10] that for maintenance of perivascular bulk flow the hydraulic parameters of the subarachnoid and perivascular spaces cannot be compromised. Although CSF losses were not observed during injections in this study, small leaks cannot be ruled out and may account for the relatively limited longitudinal displacement of ovalbumin, and the absence of statistical significance in tracer redistribution between 20 and 60 min. The apparent lack of tracer displacement between the two time points could also be explained by the possibility that these experiments were, at least partially, an investigation of spinal convection enhanced delivery. Other groups have noted that in CED studies, spinal cord injury results in migration of tracer across the grey–white border [31]. We used the smallest calibre needle possible, but there was still some evidence of local parenchymal trauma due to the cyclical movements of respiration. Unlike in Endo’s study where Evan’s blue did not cross the grey/white junction after white matter injections, tracer in this study was not completely contained within white matter at the level of injection. As with other tracer studies, labelling of the “pial glial” layer and the smooth muscle basement membrane may be explained by selective binding of tracer or by a sieving effect [8]. Fluid passage within the dorsal white columns was not directly investigated. As this area is isolated from the rest of the white matter the pattern of fluid outflow could theoretically be different. In future investigations of spinal cord fluid outflow, longer experimental time points are recommended. This would validate some of the observed differences between grey and white matter tracer distribution patterns. It may also provide insight into whether spinal CED was actually investigated in these experiments, as well as the role arterial pulsations might play in driving tracer outflow—para-venular tracer deposition may increase with time (see above). It was not possible to quantify the amount of tracer outflow via the various pathways due to the semi-quantitative nature of our results. Finally, these findings were obtained in anaesthetised prone small animals and extrapolation of these findings to upright large mammals should proceed cautiously as volatile anaesthetics are known to alter cardiovascular parameters and CSF production, which in turn affects CSF hydrodynamics [8].

## Conclusions

This study investigated the pattern and pathways of fluid outflow in the rat spinal cord. Our results suggest interstitial fluid is transported radially in the grey matter, and along the parallel axonal fibres in the white matter. Fluid outflow appears to be limited predominantly to a few spinal segments after 60 min. Paravascular and perivascular pathways, including both arterial and venous routes, likely play important roles in fluid efflux. The precise mechanisms by which the vascular basement membrane of arteries act as a conduit for fluid and solute drainage from the spinal cord warrants further investigation. There may be regional variations in fluid outflow pattern within the spinal cord due to the presence of the central canal and differences between grey and white matter in vascular anatomy. These results suggest interstitial fluid dynamics are more complicated than that described by the glymphatic model.

## Abbreviations

AFO-647: Alexa Fluor Ovalbumin 647
ASA: anterior spinal artery
CED: convection enhanced delivery
CFD: computational fluid dynamics
CNS: central nervous system
CSF: cerebrospinal fluid
ECS: extracellular space
GM: grey matter
GMi: grey matter injection
ISF: interstitial fluid
MRI: magnetic resonance imaging
NDS: normal donkey serum
PBS: phosphate buffered solution
PFA: paraformaldehyde
RECA-1: rat endothelial cell antigen-1
SAS: subarachnoid space
SEM: standard error of mean
SMA: smooth muscle antibody
WM: white matter
WMi: white matter injection

## References

[1] Coles JA, Myburgh E, Brewer JM, McMenamin PG (2017). Where are we? The anatomy of the murine cortical meninges revisited for intravital imaging, immunology, and clearance of waste from the brain. Prog Neurobiol.

[2] Brinker T, Stopa E, Morrison J, Klinge P (2014). A new look at cerebrospinal fluid circulation. Fluids Barriers CNS.

[3] Hladky SB, Barrand MA (2014). Mechanisms of fluid movement into, through and out of the brain: evaluation of the evidence. Fluids Barriers CNS.

[4] Spector R, Keep RF, Robert Snodgrass S, Smith QR, Johanson CE (2015). A balanced view of choroid plexus structure and function: focus on adult humans. Exp Neurol.

[5] Abbott NJ (2004). Evidence for bulk flow of brain interstitial fluid: significance for physiology and pathology. Neurochem Int.

[6] Hladky SB, Barrand MA (2016). Fluid and ion transfer across the blood–brain and blood–cerebrospinal fluid barriers; a comparative account of mechanisms and roles. Fluids Barriers CNS.

[7] Mokgokong R, Wang S, Taylor CJ, Barrand MA, Hladky SB (2014). Ion transporters in brain endothelial cells that contribute to formation of brain interstitial fluid. Pflugers Arch.

[8] Spector R, Robert Snodgrass S, Johanson CE (2015). A balanced view of the cerebrospinal fluid composition and functions: focus on adult humans. Exp Neurol.

[9] Rennels ML, Gregory TF, Blaumanis OR, Fujimoto K, Grady PA (1985). Evidence for a ‘paravascular’ fluid circulation in the mammalian central nervous system, provided by the rapid distribution of tracer protein throughout the brain from the subarachnoid space. Brain Res.

[10] Iliff JJ, Wang M, Liao Y, Plogg BA, Peng W, Gundersen GA, Benveniste H, Vates GE, Deane R, Goldman SA (2012). A paravascular pathway facilitates CSF flow through the brain parenchyma and the clearance of interstitial solutes, including amyloid β. Sci Transl Med.

[11] Carare RO, Bernardes-Silva M, Newman TA, Page AM, Nicoll JA, Perry VH, Weller RO (2008). Solutes, but not cells, drain from the brain parenchyma along basement membranes of capillaries and arteries: significance for cerebral amyloid angiopathy and neuroimmunology. Neuropathol Appl Neurobiol.

[12] Bilston LE, Fletcher DF, Brodbelt AR, Stoodley MA (2003). Arterial pulsation-driven cerebrospinal fluid flow in the perivascular space: a computational model. Comput Methods Biomech Biomed Eng.

[13] Cserr HF, Cooper DN, Milhorat TH (1977). Flow of cerebral interstitial fluid as indicated by the removal of extracellular markers from rat caudate nucleus. Exp Eye Res.

[14] Sykova E, Nicholson C (2008). Diffusion in brain extracellular space. Physiol Rev.

[15] Bedussi B, van Lier MG, Bartstra JW, de Vos J, Siebes M, VanBavel E, Bakker EN (2015). Clearance from the mouse brain by convection of interstitial fluid towards the ventricular system. Fluids Barriers CNS.

[16] Iliff JJ, Wang M, Zeppenfeld DM, Venkataraman A, Plog BA, Liao Y, Deane R, Nedergaard M (2013). Cerebral arterial pulsation drives paravascular CSF-interstitial fluid exchange in the murine brain. J Neurosci.

[17] Jessen NA, Munk AS, Lundgaard I, Nedergaard M (2015). The glymphatic system: a Beginner’s guide. Neurochem Res.

[18] Stoodley MA, Brown SA, Brown CJ, Jones NR (1997). Arterial pulsation-dependent perivascular cerebrospinal fluid flow into the central canal in the sheep spinal cord. J Neurosurg.

[19] Stoodley MA, Jones NR, Brown CJ (1996). Evidence for rapid fluid flow from the subarachnoid space into the spinal cord central canal in the rat. Brain Res.

[20] Wei F, Zhang C, Xue R, Shan L, Gong S, Wang G, Tao J, Xu G, Zhang G, Wang L (2017). The pathway of subarachnoid CSF moving into the spinal parenchyma and the role of astrocytic aquaporin-4 in this process. Life Sci.

[21] Endo T, Fujii Y, Sugiyama SI, Zhang R, Ogita S, Funamoto K, Saito R, Tominaga T (2016). Properties of convective delivery in spinal cord gray matter: laboratory investigation and computational simulations. J Neurosurg Spine.

[22] Milhorat THNS, Heger I, Nobandegani F, Murray S (1992). Ultrastructural evidence of sink function of central canal of spinal cord as demonstrated by clearance of horseradish peroxidase. Proc Electron Microsc Soc Am.

[23] Rossi C, Boss A, Steidle G, Martirosian P, Klose U, Capuani S, Maraviglia B, Claussen CD, Schick F (2008). Water diffusion anisotropy in white and gray matter of the human spinal cord. J Magn Reson Imaging.

[24] Brodbelt AR, Stoodley MA, Watling AM, Tu J, Jones NR (2003). Fluid flow in an animal model of post-traumatic syringomyelia. Eur Spine J.

[25] Stoodley MA, Gutschmidt B, Jones NR (1999). Cerebrospinal fluid flow in an animal model of noncommunicating syringomyelia. Neurosurgery.

[26] Wong J, Hemley S, Jones N, Cheng S, Bilston L, Stoodley M (2012). Fluid outflow in a large-animal model of posttraumatic syringomyelia. Neurosurgery.

[27] Schneider CA, Rasband WS, Eliceiri KW (2012). NIH Image to ImageJ: 25 years of image analysis. Nat Methods.

[28] Prokopova S, Vargova L, Sykova E (1997). Heterogeneous and anisotropic diffusion in the developing rat spinal cord. NeuroReport.

[29] Ellingson BM, Ulmer JL, Kurpad SN, Schmit BD (2008). Diffusion tensor MR imaging in chronic spinal cord injury. AJNR Am J Neuroradiol.

[30] Lonser RR, Gogate N, Morrison PF, Wood JD, Oldfield EH (1998). Direct convective delivery of macromolecules to the spinal cord. J Neurosurg.

[31] Wood JD, Lonser RR, Gogate N, Morrison PF, Oldfield EH (1999). Convective delivery of macromolecules into the naive and traumatized spinal cords of rats. J Neurosurg.

[32] Simonova Z, Svoboda J, Orkand P, Bernard CC, Lassmann H, Sykova E (1996). Changes of extracellular space volume and tortuosity in the spinal cord of Lewis rats with experimental autoimmune encephalomyelitis. Physiol Res.

[33] Svoboda J, Sykova E (1991). Extracellular space volume changes in the rat spinal cord produced by nerve stimulation and peripheral injury. Brain Res.

[34] Sykova E, Vargova L, Prokopova S, Simonova Z (1999). Glial swelling and astrogliosis produce diffusion barriers in the rat spinal cord. Glia.

[35] Sarntinoranont M, Chen X, Zhao J, Mareci TH (2006). Computational model of interstitial transport in the spinal cord using diffusion tensor imaging. Ann Biomed Eng.

[36] Nicholson C, Chen KC, Hrabetova S, Tao L (2000). Diffusion of molecules in brain extracellular space: theory and experiment. Prog Brain Res.

[37] Bakker EN, Bacskai BJ, Arbel-Ornath M, Aldea R, Bedussi B, Morris AW, Weller RO, Carare RO (2016). Lymphatic clearance of the brain: perivascular, paravascular and significance for neurodegenerative diseases. Cell Mol Neurobiol.

[38] Morris AW, Sharp MM, Albargothy NJ, Fernandes R, Hawkes CA, Verma A, Weller RO, Carare RO (2016). Vascular basement membranes as pathways for the passage of fluid into and out of the brain. Acta Neuropathol.

[39] Woollam DH, Millen JW (1955). The perivascular spaces of the mammalian central nervous system and their relation to the perineuronal and subarachnoid spaces. J Anat.

[40] Weller RO, Sharp MM, Christodoulides M, Carare RO, Mollgard K (2018). The meninges as barriers and facilitators for the movement of fluid, cells and pathogens related to the rodent and human CNS. Acta Neuropathol.

[41] Lam MA, Hemley SJ, Najafi E, Vella NGF, Bilston LE, Stoodley MA (2017). The ultrastructure of spinal cord perivascular spaces: implications for the circulation of cerebrospinal fluid. Sci Rep.

[42] Simon MJ, Iliff JJ (2016). Regulation of cerebrospinal fluid (CSF) flow in neurodegenerative, neurovascular and neuroinflammatory disease. Biochim Biophys Acta.

[43] Asgari M, de Zélicourt D, Kurtcuoglu V (2016). Glymphatic solute transport does not require bulk flow. Sci Rep.

[44] Jin BJ, Smith AJ, Verkman AS (2016). Spatial model of convective solute transport in brain extracellular space does not support a “glymphatic” mechanism. J Gen Physiol.

[45] Smith AJ, Yao X, Dix JA, Jin BJ, Verkman AS (2017). Test of the ‘glymphatic’ hypothesis demonstrates diffusive and aquaporin-4-independent solute transport in rodent brain parenchyma. Elife.

[46] Abbott NJ, Pizzo ME, Preston JE, Janigro D, Thorne RG (2018). The role of brain barriers in fluid movement in the CNS: is there a ‘glymphatic’ system?. Acta Neuropathol.

[47] Hawkes CA, Jayakody N, Johnston DA, Bechmann I, Carare RO (2014). Failure of perivascular drainage of beta-amyloid in cerebral amyloid angiopathy. Brain Pathol.

[48] Weller RO, Djuanda E, Yow HY, Carare RO (2009). Lymphatic drainage of the brain and the pathophysiology of neurological disease. Acta Neuropathol.

[49] Arbel-Ornath M, Hudry E, Eikermann-Haerter K, Hou S, Gregory JL, Zhao L, Betensky RA, Frosch MP, Greenberg SM, Bacskai BJ (2013). Interstitial fluid drainage is impaired in ischemic stroke and Alzheimer’s disease mouse models. Acta Neuropathol.

[50] Morrison PF, Laske DW, Bobo H, Oldfield EH, Dedrick RL (1994). High-flow microinfusion: tissue penetration and pharmacodynamics. Am J Physiol.

[51] Ichimura T, Fraser PA, Cserr HF (1991). Distribution of extracellular tracers in perivascular spaces of the rat brain. Brain Res.

[52] Schley D, Carare-Nnadi R, Please CP, Perry VH, Weller RO (2006). Mechanisms to explain the reverse perivascular transport of solutes out of the brain. J Theor Biol.

[53] Stoodley MA, Jones NR, Yang L, Brown CJ (2000). Mechanisms underlying the formation and enlargement of noncommunicating syringomyelia: experimental studies. Neurosurg Focus.

[54] Murtha LA, Yang Q, Parsons MW, Levi CR, Beard DJ, Spratt NJ, McLeod DD (2014). Cerebrospinal fluid is drained primarily via the spinal canal and olfactory route in young and aged spontaneously hypertensive rats. Fluids Barriers CNS.

